# A multicentre randomised controlled trial evaluating lactobacilli and bifidobacteria in the prevention of antibiotic-associated diarrhoea in older people admitted to hospital: the PLACIDE study protocol

**DOI:** 10.1186/1471-2334-12-108

**Published:** 2012-05-06

**Authors:** Stephen J Allen, Kathie Wareham, Caroline Bradley, Wyn Harris, Anjan Dhar, Helga Brown, Alwyn Foden, Way Yee Cheung, Michael B Gravenor, Sue Plummer, Ceri J Phillips, Dietrich Mack

**Affiliations:** 1 Swansea University, Singleton Park, Swansea, SA2 8PP, UK; 2 Clinical Research Unit, Morriston Hospital, Swansea SA6 6NL, UK; 3 County Durham and Darlington NHS Foundation Trust, Darlington Memorial Hospital, Hollyhurst Road, Darlington, County Durham DL3 6HX, UK; 4 Abertawe Bro Morgannwg University Hospital Board, One Talbot Gateway, Baglan Energy Park, Baglan, Port Talbot SA12 7BR, UK; 5 Obsidian Research Limited, Unit 2 Christchurch Road, Baglan Industrial Park, Port Talbot, West Glamorgan SA12 7BZ, UK; 6 The College of Medicine, Swansea University, Room 314, Grove Building, Singleton Park, Swansea SA2 8PP, UK

**Keywords:** Probiotic, Lactobacilli, Bifidobacteria, Antibiotic, Antibiotic associated diarrhoea, *Clostridium difficile* diarrhoea, Elderly, Hospital in-patient

## Abstract

**Background:**

Antibiotic associated diarrhoea complicates 5–39% of courses of antibiotic treatment. Major risk factors are increased age and admission to hospital. Of particular importance is *C. difficile* associated diarrhoea which occurs in about 4% of antibiotic courses and may result in severe illness, death and high healthcare costs. The emergence of the more virulent 027 strain of *C. difficile* has further heightened concerns. Probiotics may prevent antibiotic associated diarrhoea by several mechanisms including colonization resistance through maintaining a healthy gut flora.

**Methods:**

This study aims to test the hypothesis that administration of a probiotic comprising two strains of lactobacilli and two strains of bifidobacteria alongside antibiotic treatment prevents antibiotic associated diarrhoea. We have designed a prospective, parallel group trial where people aged 65 years or more admitted to hospital and receiving one or more antibiotics are randomly allocated to receive either one capsule of the probiotic or a matching placebo daily for 21 days. The primary outcomes are the frequency of antibiotic associated and *C. difficile* diarrhoea during 8–12 weeks follow-up. To directly inform routine clinical practice, we will recruit a sufficient number of patients to demonstrate a 50% reduction in the frequency of *C. difficile* diarrhoea with a power of 80%. To maximize the generalizability of our findings and in view of the well-established safety record of probiotics, we will recruit a broad range of medical and surgical in-patients from two different health regions within the UK.

**Discussion:**

Antibiotic associated diarrhoea constitutes a significant health burden. In particular, current measures to prevent and control *C. difficile* diarrhoea are expensive and disrupt clinical care. This trial may have considerable significance for the prevention of antibiotic associated diarrhoea in hospitals.

**Trial registration:**

International Standard Randomised Controlled Trial Number Register ISRCTN70017204.

## Background

Antibiotic-associated diarrhoea (AAD) is diarrhoea that occurs in association with antibiotic treatment and without an alternative cause [1]. It is estimated to occur in 5 – 39% of people taking antibiotics during or up to 12 weeks after treatment [2]. AAD varies markedly in severity from mild diarrhoea to severe diarrhoea as part of a life-threatening illness caused by the anaerobic bacterium *Clostridium difficile* (*Clostridium difficile* associated diarrhoea*;* CDAD) which accounts for 15–39% of cases of AAD [3]. A particular concern is the emergence of the hypervirulent ribotype 027 strain of *C. difficile* documented in Canada, USA and Europe [4]. Even mild diarrhoea may cause significant morbidity and prolong hospital stay. The annual healthcare cost of CDAD in the USA was estimated to be more than $3 billion [5,6].

Host risk factors for AAD include advanced age, immune suppression, gastrointestinal disease or previous gastrointestinal surgery and severe co-morbidity [2,3,7]. AAD can complicate treatment with any antibiotic but broad spectrum antibiotics (clindamycin, cephalosporins, β-lactamase resistant penicillins, quinolones), antibiotic combinations and repeated or long treatment courses are associated with the greatest risk of AAD [2,7]. Other environmental factors include admission to hospital, longer duration of stay and use of naso-gastric tubes [8]. Suppression of gastric acid may also be an additional risk factor for CDAD in both hospital inpatients [9] and people in the community [10].

Antibiotics may result in diarrhoea through several mechanisms. Antibiotic disruption of the commensal gut microorganisms results in osmotic or secretory diarrhoea from changes in carbohydrate, short chain fatty acid and bile acid metabolism and impaired colonization resistance allowing a competitive growth advantage to diarrhoeagenic organisms [11-13].

Given the frequency and potential severity of CDAD, extensive and costly strategies for the prevention of AAD have become an established part of hospital in-patient management. These include strict limitations on the use of antibiotics, hand washing, isolation of diarrhoea patients and decontamination of the environment [14]. However, despite these efforts, the heat- and drying-resistant spores of *C. difficile* persist in the modern hospital environment [15,16].

In view of the mechanisms underlying AAD, probiotics offer a potential means of prevention. Probiotics are defined as live microbial organisms which, when administered in adequate numbers, are beneficial to health [17]. In a recent meta-analysis, McFarland [18] pooled data from 25 RCTs (total of 2,810 adults and children) and reported a reduced relative risk of AAD in participants receiving a probiotic (0.43; 95% CI 0.31 – 0.58). Trials of many different single strains of probiotic bacteria, the yeast *Saccharomyces boulardii (S. boulardii*)*,* probiotic mixtures and probiotic and prebiotic mixtures were included in this analysis. Dosage (number of organisms) also varied markedly between studies. In sub-group analyses, factors associated with greater efficacy in preventing AAD were use of *S. boulardii* or *Lactobacillus rhamnosus (L. rhamnosus) GG*, mixtures of probiotics and preparations with high numbers of organisms.

Members of our research group [19] assessed the effect of a combination of *L. acidophilus* and *B. bifidum* on CDAD in a pilot study in elderly patients receiving antibiotics. Overall, 30/138 (22%) patients developed diarrhoea with 5/69 in the placebo group and 2/69 in the probiotic group testing positive for *C. difficile* toxin. In this small study, the main effect of the intervention appeared to be neutralisation of the toxin rather than prevention of colonization with *C. difficile*. The meta-analysis by McFarland [18] included 5 studies, in addition to our study, but all of these were treatment trials of patients with established or recurrent CDAD. When designing our study, apart from a small trial with few CDAD cases [20], we were not aware of other studies that had assessed probiotics in the primary prevention of CDAD in hospitalized adults. Since then, reports of trials have been consistent with the prevention of CDAD by lactobacilli and bifidobacteria although relatively small numbers of participants were recruited [21,22].

Probiotics were classified by the US Food and Drug Administration in 2002 (notice GRN 000049) as “generally regarded as safe”. Reported adverse events in research studies were mild but occurred with *S. boulardii* (constipation, increased thirst) and *L. rhamnosus* GG (bloating, gas) [18]. Probiotics have been administered without adverse effects to vulnerable groups such as children and adults with HIV infection and preterm infants [23]. However, an expert group in the USA concluded recently that although the available evidence from randomised trials did not indicate a widespread risk of adverse events, researchers should monitor and quantify adverse events in future probiotic intervention studies [24].

In summary, a variety of probiotics appear to reduce the risk of AAD. However, the large number of different probiotics and different regimens that have been evaluated compromises the development of clinical practice guidelines. Also, many trials have included only a small number of participants, trial methods have often been poor, the period of follow-up limited and the quality control of the probiotic interventions has been inadequate [25]. There are insufficient data to assess the effectiveness of probiotics in the prevention of CDAD.

## Presentation of the hypothesis

In older people admitted to hospital and treated with one or more antibiotics, administration of a multi-strain probiotic would reduce the frequency of AAD and CDAD.

## Testing the hypothesis

This study is a double-blind, randomized, parallel group, placebo-controlled trial. We plan to recruit patients with a wide range and severity of illness from as many wards as possible in 5 secondary care hospitals from 2 geographically distant UK health authorities, in order to be confident that our findings are applicable to a diverse range of older patients admitted to National Health Service (NHS) hospitals and similar institutions. Planned recruitment sites in the Abertawe Bro Morgannwg University Health Board (ABMUHB), South West Wales are Singleton and Morriston hospitals (total 1450 beds), and in the County Durham and Darlington Foundation NHS Trust (CDDFT), University Hospital of North Durham, Bishop Auckland General Hospital and Darlington Memorial Hospital (1353 beds). In 2005/6 (12 months), 26,692 people aged ≥65 years were admitted in Swansea and 21,676 in CDDFT and approximately one third received antibiotics.

Inclusion and exclusion criteria are shown in Table 1. Exclusion criteria will be limited to possible contraindications to probiotics identified in previous studies and case reports [23]. In particular, following the findings of the PROPATRIA trial [26], we will exclude patients with severe illness requiring high dependency or intensive care (but not planned admission to these facilities for observation only), acute pancreatitis, any known abnormality or disease compromising intestinal blood supply, and those with a jejunal tube *in situ* or receiving jejunal feeds .

**Table 1:** Criteria for participant inclusion and exclusion

	· people aged ≥65 years and admitted to hospital
**Inclusion criteria**	· exposed to one or more antibiotics within the last 7 days or are about to start antibiotic treatment
	· consultant approval to invite patient to join the study
	· diarrhoea present^1^
	· immunocompromise^2^
	· severe illness requiring high dependency or intensive care^3^
	· prosthetic heart valve
	· *C. difficile* in past 3 months
**Exclusion criteria**	· active inflammatory bowel disease^4^
	· suspected acute pancreatitis^5^
	· known compromised gut blood supply^6^
	· jejunal tube *in situ* or receiving jejunal feeds
	· previous adverse reaction to probiotics
	· unwilling to discontinue exiting use of probiotics

^1^Defined as 3 or more watery or loose stools (Bristol Stool Form Scale types 5–7) in a 24 h period. ^2^Sufficient to require isolation and barrier nursing. ^3^Planned admission to these facilities for observation only is not an exclusion criteria. ^4^Required specific treatment in past 12 months. ^5^Abdominal pain with serum amylase/lipase ≫ ×3 institutional upper limit of normal. ^6^Abnormality or disease of mesenteric vessels or coeliac axis.

The investigational medicinal products (IMPs) will be discontinued and participants withdrawn from the study if they develop pancreatitis or severe illness that requires high dependency or intensive care. However, these participants will be included in intention-to-treat analysis.

**Figure 1:**
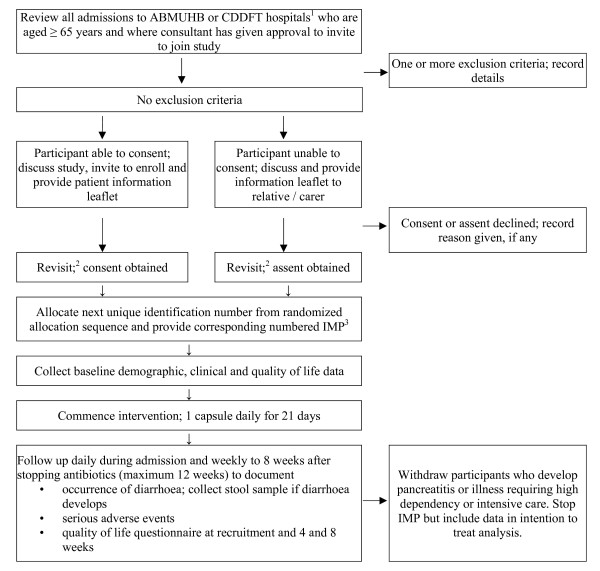
**Participant flow chart.** Notes: 1. ABMUHB – Abertawe Bro Morgannwg University Health Board; CDDFT – County Durham and Darlington NHS Foundation Trust. 2. The patient or next of kin is approached for consent in the afternoon if verbal and written information about the trial is provided in the morning, or the following day if provided in the afternoon. 3. Investigational medicinal product; either 21 capsules of probiotic or placebo.

The flow chart regarding screening, recruiting and following-up participants is shown in Figure 1. A research nurse will take signed, informed consent according to standard guidelines [27]. It is expected that some potential participants will not be able to give informed consent because of impaired mental capacity. Assent will be sought from next of kin, other relatives or carers in line with Article 5 of the EU Directive 2001/20/EC (Clinical Trials on incapacitated adults not able to give informed legal consent). The consent form will be held in the investigator file, with copies filed in the hospital notes and given to the participant. A sticker will be placed on the hospital notes and the patient’s General Practitioner informed by letter. The reasons for declining to participate, if provided, will be recorded.

Demographic and clinical data will be recorded at recruitment and include the type and dose of antibiotics and other potential risk factors for AAD or CDAD (Table 2).

**Table 2:** Baseline demographic and clinic data and risk factors for AAD

**Variable**	**Units or Categories where appropriate**
· Age	years
· Sex	male; female
· Race	white; black; Asian; Chinese; other
· Period of recruitment	summer (May – Sept); winter (Oct-March)
· Smoking	usual number of cigarettes/day
· Alcohol	usual number of units/week
· Where admitted from	home; residential care; other hospital; other
· Admission type	emergency/unplanned; elective/ planned for procedure or investigation
· Admission diagnosis or main cause of admission	
· Co-morbidity	hypertension; asthma; diabetes; chronic obstructive airways disease; renal disease; irritable bowel syndrome; dementia; Alzheimer’s disease; other
· Previous GI surgery	
· Number of hospital admissions in last eight weeks	
· Antacid treatment	antacid; H_2_-receptor antagonist; proton pump inhibitor
· Naso-gastric tube *in-situ*	
· Duration of hospital stay prior to recruitment	days
· Indication for antibiotic treatment	
· Antibiotic treatment: antibiotic used^1^	single class; 2 classes; 3 or more classes
· Antibiotic treatment: total duration	single dose; 1–6 days; 7–13 days; ≥14 days

^1^Antibiotics will be classified according to British National Formulary categories (http://www.bnf.org): Penicillins (sub-classified as benzylpenicillin, penicillinase resistant penicillin, broad-spectrum penicillins, anti-pseudomonas penicillins); Cephalosporins (sub-classified as 1^st^, 2^nd^ and 3^rd^ generation); Carbapenems and other beta-lactams; Tetracyclines; Aminoglycosides; Macrolides; Clindamycin; Sulphonamides and trimethoprim; Metronidazole; Quinolones; Glycopeptides; TB drugs; other.

### Generation and concealment of a random allocation sequence

A random allocation sequence using blocks of variable sizes will be produced using SAS PROC PLAN Version 9.1 (SAS Institute Inc., Cary, NC, USA). The sequence will allocate participants to either placebo or probiotic on a 1:1 basis and be stratified by centre to ensure that similar numbers of patients will be allocated to each arm of the study in each centre. The allocation sequence will not be available to members of the research team during participant recruitment and follow-up.

### Investigational medicinal products and participant allocation to trial arms

The absence of a scientific rationale for selecting a particular strain and dosage of organisms for specific health outcomes is reflected in the many different probiotic preparations evaluated in the prevention of antibiotic-associated diarrhea [28]. However, the use a combination of different organisms with large numbers of each strain is likely to maximize gut colonization and, thereby, colonization resistance. To minimise the risk of adverse effects such as systemic infection, we decided not to consider *S. boulardii* or other organisms that are not part of the normal human commensal flora.

Based on evidence of ability to survive passage through the upper gut, adherence to intestinal mucosa, excellent viability at the point of administration, our earlier study [19], and evidence that *L. acidophilus* neutralises *C. difficile* toxin in an epithelial cell assay *in vitro* (SP pers. comms), we selected a probiotic comprised of two strains of *L. acidophilus* (CUL60, National Collection of Industrial, Food and Marine Bacteria [NCIMB] 30157 and CUL21, NCIMB 30156), *Bifidobacterium bifidum* (*B. bifidum*; CUL20, NCIMB 30153) and *B. lactis* (CUL34, NCIMB 30172). The probiotic is prepared as lyophilised powder in a vegetarian capsule containing a total of 6 x 10^10^ organisms/capsule. These organisms are commercially available through BioCare, UK and Pharmax, USA. The placebo is an identical capsule containing inert maltodextrin powder. The dosage is 1 capsule/day taken with food and, where possible, between antibiotic doses, for 21 days.

To comply with labeling requirements, Obsidian Research Ltd., UK will prepare bottles labeled with each unique study number in the series and containing 21 capsules of the appropriate IMP according to the random allocation sequence. The research nurse will allocate each participant sequentially to the next unique study number in the sequence and provide them with the corresponding IMP bottle. The participant will be instructed to take the first dose of the IMP on the day of recruitment. The daily administration of the IMP will be supervised by the research nurses in ABMUHB and by the ward nurses in CDDFT during hospital admission and participants will be encouraged to complete the course once discharged.

The process for acquiring a license for provision of the IMPs was begun in March 2008 and a Medicines and Healthcare products Regulatory Agency (MHRA) inspector notified in April 2008. The MHRA inspected the facility responsible for the packaging and quality control of the IMPs and the license was granted in November 2008. We had underestimated the time required for MHRA approval and this resulted in a delay in starting participant recruitment.

To ensure the quality of the probiotic, organism identity will be checked by established molecular techniques and viability by quantitative bacterial culture in study preparations retrieved from wards during the study. This will be done by an independent laboratory to maintain masking of the allocation sequence. *In vitro* antibiotic testing demonstrated that the probiotic bacteria were generally sensitive to broad spectrum antibiotics and all four strains were sensitive to ceftriaxone, chloramphenicol, erythromycin, linezolid and tetracycline.

### Follow-up

Research nurses will review participants daily during admission to determine the onset, frequency and duration of any diarrhoea and ask about gastrointestinal symptoms (abdominal pain, bloating, flatus, nausea), acceptability and adverse effects of the interventions. After discharge, follow-up will be weekly by telephone call, postal questionnaire or home visit as appropriate. Participants will be provided with contact details for ready telephone access to research staff to notify the onset of diarrhoea or adverse events. All participants will be followed-up for 8 weeks after completing antibiotic treatment to a maximum of 12 weeks from recruitment for those on longer antibiotic courses.

### Outcome measures

The two outcomes used to generate the sample size, AAD and CDAD, have been identified as co-primary outcomes (Table 3). Diarrhoea is defined as 3 or more loose stools in a 24 h period. We will use the Bristol Stool Form Scale [29] to assist participants in describing stool consistency; types 5-7 will be consistent with diarrhoea.

**Table 3:** Outcome measures

**Primary outcomes**	1. the occurrence of AAD
	2. the occurrence of CDAD
	1. severity^1^ and duration of AAD
	2. severity^1,2^ and duration of CDAD and incidence of recurrence within the 8-12 weeks follow-up period
	3. abdominal symptoms (abdominal pain, bloating, flatus, nausea)
**Secondary outcomes**	4. well-being and quality of life assessed using the generic measures EQ-5D and the York SF12 (Iglesias 2001) at recruitment and 4 and 8 weeks
	5. duration of hospital stay
	6. serious adverse effects
	7. acceptability of the probiotic preparation
	8. viability of the probiotic at point of administration
	9. cost-effectiveness

^1^Frequency and consistency of stools. ^2^Additional information extracted from the clinical records (*e.g.* laboratory parameters, findings at sigmoidoscopy, pseudomembraneous colitis, colectomy, death).

All participants who develop diarrhoea during the study period will be asked to provide a stool sample and this will be collected during a home visit if required. The cause of diarrhoea will be determined by NHS laboratories according to their usual practice. Stools will be analysed for diarrhoeal pathogens (bacterial culture for *Salmonella* sp, *Shigella* sp, Campylobacter, *E. Coli* 0157; wet film for ova, cysts and parasites) and for *C. difficile* toxins. In ABMUHB, *C. difficile* will be identified by an in-house culture assay with further analysis by Meridian Premier Toxin A + B Enzyme Immunoassay (Meridian Bioscience Europe Inc., Cincinnati, USA) if required. In CDDFT, toxins are identified using the mini VIDAS system (C. diff Quik Check, Techlab, Blacksburg, U.S.A.). If a cause of the diarrhoea is not identified, a further stool sample will be collected and tested 2 days later.

For the primary outcome measures, AAD is defined as diarrhoea without pathogens detected on routine laboratory analysis, negative for *C. difficile* toxin and without alternative cause (e.g. laxative treatment). CDAD is defined as diarrhoea with stools positive for *C. difficile* A or B toxin. For quality control purposes, *C. difficile* culture and confirmation by immunoassay will be undertaken in 1 in 5 *C. difficile* toxin positive stool samples collected in Swansea. In view of the known inadequacies of current methods for the laboratory diagnosis of *C. difficile *[30], we will also save stool samples at −20°C from participants with diarrhoea for further testing for the glutamate dehydrogenase antigen and also lactoferrin (IBD scan and C. diff Quik Chek Complete; Alere Limited, Stockport, UK). This may result in the diagnosis of CDAD that was not detected by routine methods.

Participants who develop severe diarrhoea will be investigated and managed according to the current practice of their clinicians. We will extract information from the clinical records (*e.g.* laboratory parameters, findings at sigmoidoscopy, colectomy) to classify the severity of CDAD. At the end of follow-up, participants will be classified as either developing AAD, CDAD, another cause of diarrhoea (*e.g.* norovirus) or no diarrhoea. Other endpoints have been listed as secondary outcomes (Table 3).

### Sample size and recruitment

Conservatively, we expected ADD to occur in 20% and CDAD in 4% of participants allocated to the placebo group [2]. To detect a 50% reduction in the frequency CDAD in the probiotic group (i.e. 2% frequency) with 80% power at the 5% significance level, would require 2,478 subjects (1,239 in each group; 1:1 allocation). At the 5% significance level, this number of participants would provide a power of ≫99% to detect a 50% reduction in ADD (i.e. 10% frequency) and a power of 90% to detect a 25% reduction in ADD (i.e. 15% frequency) in the probiotic group. To allow for 10% drop-outs and 10% loss to follow-up due to deaths unrelated to diarrhoea, we plan to recruit 2,974 participants.

Based on data from 2005/6, about 14,000 patients per year aged ≥65 years and exposed to antibiotics were admitted to the study hospitals and there was a total of 963 cases of CDAD. Conservatively, we expect to be able to recruit between 1:9 and 1:10 of eligible patients (approximately 124 patients/month) and undertake recruitment for a total of 24 months. This estimate was supported by a 2-week pilot study of the recruitment process in 23 medical and surgical wards in Morriston Hospital when research nurses visited the wards daily. Out of a total of 253 admissions aged ≥65 years, 166 patients were excluded (152 patients had no current or planned exposure to antibiotics; 12 already had diarrhoea [including 1 CDAD]; 1 active inflammatory bowel disease; 1 reported a previous adverse reaction to a probiotic). Eighty-seven (34.4%) patients were eligible to participate in the pilot study but 8 of these were either confused or not available (in theatre or undergoing investigations). Therefore, the study design was explained to 79 patients (31.2% of total admissions) and 58 (73.4%) stated that they would have agreed to participate in this study.

We will monitor the number of participants reaching study endpoints in each hospital every 3 months. The ethical permission secured for the study included all hospitals within each health authority providing an ability to extend recruitment if needed.

### Statistical analysis

Primary outcomes will be analysed with standard methods for a binary outcome. Risk difference and odds ratio between two groups and their 95% confidence intervals for AAD and CDAD will be estimated. In addition, covariate adjustment analysis that includes the relevant covariates (such as age, gender, specific antibiotic, centre) will be performed. To explore whether prevention strategies should be provided to all patients or just those at high risk of diarrhoea, we will assess probiotic efficacy according to known or potential risk factors for AAD and CDAD as described previously. Sub-group effects will be identified by inspection of interaction terms, and these will be interpreted in light of power relative to main effects and supporting evidence of mechanism [31]. All analyses will be performed using SAS PROC PLAN Version 9.1 (SAS Institute Inc., Cary, NC, USA).

There are few tools that are validated for measuring QoL in older people and none specifically targeted at treatment-induced diarrhoea. We will use the generic measures EQ-5D and the York SF12 [32] to understand the broader health impact related to treatment-induced diarrhoea and facilitate cost-effectiveness analysis.

Health economic analysis will be undertaken from the perspective of the UK NHS. Resources utilised by each participant will include the cost of the probiotics, staff time involved in administering probiotics, treatment of adverse events, assessment of cases of diarrhoea (stool collection and culture/toxin assay, endoscopy) and the costs of dealing with and treating diarrhoea such as laundry, antibiotics, increased hospital stay and co-morbidities. Unit costs will be determined from discussion with relevant clinicians and finance department staff as well as from recognised published information.

Cost differences between the probiotic and placebo group will be determined and used in conjunction with differences in outcomes between groups in undertaking a cost-consequences analysis. Cost per case of diarrhoea averted will be the primary outcome measure. Sub-group analyses will determine the relative cost-effectiveness of preventative strategies in different risk groups. In addition, a cost-utility analysis will be undertaken based on the differences in costs between the two groups and in quality-adjusted life years derived from responses to the EQ-5D questionnaire.

Given the short timescale of the project, there will be no discounting of the costs or benefits. Sensitivity analyses will investigate the robustness of the results to changes in estimated costs and outcomes and probabilistic sensitivity analysis will use bootstrap resampling to determine the probability that preventive strategies are within certain thresholds. The budgetary impact from a UK NHS perspective of adopting a policy of administering a probiotic preparation to prevent or ameliorate AAD and CDAD in people aged 65 years and over admitted to secondary care NHS facilities and receiving oral or intravenous antibiotics will also be assessed.

### Trial organisation and safety monitoring

The funders (the National Institute of Health Research Health Technology Assessment [HTA] Programme) will appoint the chairs and members for a Trial Steering Committee (TSC) and an independent Data Monitoring and Ethics Committee (DMEC). The DMEC will include an independent statistician who will generate and hold the random allocation sequence for the trial. Trial management will be centered in the Clinical Research Unit, Morriston Hospital, Swansea. A Trial Management Committee will include the Principle Investigator, the Project Manager, the CDDFT Site Co-ordinator and CDDFT hospital site leads.

In view of the expected high frequency of adverse events in an elderly population of hospital in-patients, the recording of gastro-intestinal symptoms during follow-up and the excellent safety record of probiotics, only information regarding serious adverse events (SAEs) will be recorded. SAEs will be defined and reported according to GCP guidelines [27], assessed by the local research clinicians involved in the project as to their attribution and reviewed regularly by an Independent Safety Monitor.

Suspected Unexpected Serious Adverse Reactions (SUSARs) will be defined as the development of:

1. bacterial infection (any manifestation of infection: abscess, bacterial endocarditis, bacteraemia etc.) where a lactobacillus or bifidobacteria is isolated in pathological specimens

2. multi-organ failure

3. bowel ischaemia

4. pancreatitis (abdominal pain with serum amylase or lipase concentration ≥3 times the institutional upper limit of normal)

SUSARs will be reported immediately to the Independent Safety Monitor to consider their attribution to the participant’s inclusion in the trial. It was considered that knowledge of a participant’s allocation would not inform the clinical management of an SAE or SUSAR and, therefore, procedures for the immediate unmasking of participant allocation will not be established. If required, unmasking could be undertaken by the Independent Statistician. The Independent Statistician will undertake an unblinded, interim analysis of SAEs and SUSARs in the first 500 participants with complete data and report the findings to the DMEC.

## Implications of the hypothesis

Current evidence is insufficient to inform the use of probiotics in the prevention of AAD and CDAD in routine clinical practice. Recommendations for substantiating the health effects of probiotics have been published [33,34]. We plan to evaluate a multi-strain probiotic consisting of two strains of lactobacilli and two strains of bifidobacteria in the prevention of AAD. We plan to recruit a large number of patients admitted to hospitals and receiving antibiotics in the UK that will be representative of the population of hospitalized older patients in industrialised countries. Causes of diarrhoea will be assessed according to usual practice to investigate the effectiveness and cost-effectiveness of this intervention if it were implemented into routine practice.

The proposed study received ethics approval from the Research Ethics Committee for Wales in November 2008 and from the NHS Research and Development Departments responsible for the hospital sites. Recruitment of participants began in June 2009 in ABMUHB and October 2009 in CDDFT and is estimated to be completed by March 2012. Published results are expected later in 2012.

## Abbreviations

AAD = Antibiotic associated diarrhoea; ABMUHB = Abertawe Bro Morgannwg University Health Board; B. bifidum = Bifidobacterium bifidum; B. lactis = Bifidobacterium lactis; CDAD = Clostridium difficile associated diarrhoea; CDDFT = County Durham and Darlington NHS Foundation Trust; DMEC = Data Monitoring and Ethics Committee; GCP = Good Clinical Practice; HTA = Health Technology Assessment; ICH = International Conference on Harmonisation; IMP = Investigational medicinal product; L. acidophilus = Lactobacillus acidophilus; MHRA = Medicines and Healthcare products Regulatory Agency; NCIMB = National Collection of Industrial, Food and Marine Bacteria; NHS = National Health Service; PLACIDE = Probiotic lactobacilli and bifidobacteria in antibiotic-associated diarrhoea and Clostridium difficile diarrhoea in the elderly; S. boulardii = Saccharomyces boulardii; SAE = Severe adverse event; SUSAR = Suspected unexpected serious adverse reaction; TSC = Trial Steering Committee.

## Competing interests

Stephen Allen has undertaken research in probiotics supported by Cultech Ltd, Baglan, Port Talbot, UK, been an invited guest at the Yakult Probiotic Symposium in 2011 and received research funding from Yakult, UK in 2010. Sue Plummer is a Director of Cultech Ltd. UK. The remaining authors declare that they have no competing interests.

## Authors’ contributions

SA conceived of the study. All authors were involved in the design of this study and all have contributed to and approved the final manuscript.

## Authors’ information

SA; Professor of Paediatrics and International Health; Honorary Consultant Paediatrician. KW; Director, Clinical Research Unit. CB; Formally Lead Clinical Pharmacist, Antibiotic Use and Clinical Governance. WH; Consultant Physician/Geriatrician. AD; Consultant Gastroenterologist. HB; Consultant Physician. AF; Consultant Chest Physician. WYC; Senior Lecturer, Health Services Research. MG; Professor of Biostatistics and Epidemiology. SP; Technical Director. CJP; Professor of Health Economics. DM; Professor of Medical Microbiology and Infectious Diseases

## Pre-publication history

The pre-publication history for this paper can be accessed here:

http://www.biomedcentral.com/1471-2334/12/108/prepub
